# Ethnobotanical, Phytochemical, Toxicological, and Pharmacological Properties of *Ziziphus lotus* (L.) Lam.: A Comprehensive Review

**DOI:** 10.3390/ph16040575

**Published:** 2023-04-11

**Authors:** Noureddine Bencheikh, Fatima Zahrae Radi, Jamila Fakchich, Amine Elbouzidi, Sabir Ouahhoud, Mohammed Ouasti, Mohamed Bouhrim, Imane Ouasti, Christophe Hano, Mostafa Elachouri

**Affiliations:** 1 Laboratory of Bioresources, Biotechnology, Ethnopharmacology and Health, Faculty of Sciences, Mohammed First University, Boulevard Mohamed VI, B.P. 717, Oujda 60000, Morocco; 2 Research Team of Chemistry of Bioactive Molecules and the Environment, Laboratory of Innovative Materials and Biotechnology of Natural Resources, Faculty of Sciences, Moulay Ismail University of Meknes, B.P. 11201, Zitoune, Meknes 50070, Morocco; 3 Laboratoire d’Amélioration des Productions Agricoles, Biotechnologie et Environnement (LAPABE), Faculté des Sciences, Université Mohammed Premier, Oujda 60000, Morocco; 4 Laboratory of Biological Engineering, Team of Functional and Pathological Biology, Faculty of Sciences and Technology Beni Mellal, University Sultan Moulay Slimane, Beni-Mellal 23000, Morocco; 5 Laboratoire de Biologie des Ligneux et des Grandes Cultures, INRAE USC1328, Campus Eure et Loir, Orleans University, 28000 Chartres, France

**Keywords:** *Ziziphus lotus* (L.) Lam., ethnobotany, traditional medicine, phytochemical, toxicological, pharmacological activities

## Abstract

*Ziziphus lotus* (L.) Lam. (Rhamnaceae) is a plant species found across the Mediterranean area. This comprehensive overview aims to summarize the botanical description and ethnobotanical uses of *Z. lotus* and its phytochemical compounds derived with recent updates on its pharmacological and toxicological properties. The data were collected from electronic databases including the Web of Science, PubMed, ScienceDirect, Scopus, SpringerLink, and Google Scholars. It can be seen from the literature that *Z. lotus* is traditionally used to treat and prevent several diseases including diabetes, digestive problems, urinary tract problems, infectious diseases, cardiovascular disorders, neurological diseases, and dermal problems. The extracts of *Z. lotus* demonstrated several pharmacological properties in vitro and in vivo such as antidiabetic, anticancer, anti-oxidant, antimicrobials, anti-inflammatory, immunomodulatory, analgesic, anti-proliferative, anti-spasmodic, hepatoprotective, and nephroprotective effects. The phytochemical characterization of *Z. lotus* extracts revealed the presence of over 181 bioactive compounds including terpenoids, polyphenols, flavonoids, alkaloids, and fatty acids. Toxicity studies on *Z. lotus* showed that extracts from this plant are safe and free from toxicity. Thus, further research is needed to establish a possible relationship between traditional uses, plant chemistry, and pharmacological properties. Furthermore, *Z. lotus* is quite promising as a medicinal agent, so further clinical trials should be conducted to prove its efficacy.

## 1. Introduction

It is well-known that plants have been used in popular medicine since ancient times to treat and protect against a variety of human diseases [1,2]. According to the World Health Organization, the percentage of people using traditional medicine including plants is estimated to be 80% [3]. The security, efficacy, economic feasibility, and accessibility are the most important criteria justifying these traditional practices [4,5]. Recently, the traditional uses of medicinal herbs in drug therapies have grown in importance. The therapeutic efficacy of these herbs is mainly due to the presence of secondary metabolites that have interesting biological properties [6]. In fact, more than 60% of approved pharmaceutical drugs are obtained from natural sources, notably vegetation matter [7]. The use of medicinal herbs in drug therapies has grown in importance, either directly or as a crude resource for separating chemical compounds with particular bioactivities [6]. Among a range of medicinal plants, the genus *Ziziphus*, belonging to the Rhamnaceae family, is frequently utilized in traditional medicine to cure and alleviate health problems [2,8,9,10]. The genus *Ziziphus* includes 58 accepted plant species, according to World Flora Online (WFO). Among the species of this kind, *Ziziphus lotus* (L.) Lam. (*Z. lotus*) is widely used in different countries, particularly in North African countries, for treating various ailments. This plant, native to the Mediterranean region, is widely distributed in Africa as well as other areas in the Asian region (China, Iran, and South Korea) [11,12,13]. This plant species is traditionally used for treating and preventing many diseases such as diabetes, digestive system problems, urinary tract problems, contagious diseases, cardiovascular diseases, neuronal diseases, and dermal problems. Several published pharmacological studies have indicated that the extracts from various parts of *Z. lotus* have significant bioactivities such as antioxidant, antimicrobial, hepato-nephroprotective, antihyperlipidemic, anti-inflammatory, analgesic, and antiproliferative effects [14,15,16,17,18,19,20,21,22]. Furthermore, the assessment of the toxicity of natural products including derivatives of plant matter is necessary for the manufacturing process of pharmaceutical products. Toxicological investigations on *Z. lotus* extracts indicate that this plant does not represent any risk of toxicity [22,23]. Several phytochemical studies have indicated that *Z. lotus* extracts are abundant in natural substances such as polyphenols, flavonoids, terpenes, and phenolic acids [20,21,22,23,24,25,26,27]. These phytochemical compounds are well-known in the scientific literature for their pharmacological effects against various pathologies.

Abdoul-Azize (2016) conducted a literature review on the potential nutritional and health benefits of bioactive jujube (*Z. Lotus*) compounds [28]. This review collected and analyzed approximately 79 references on *Z. lotus* up to 2016. However, this bibliographic study focused on the phytochemistry and nutritional aspects of *Z. lotus*, and does not go into detail on the plant’s pharmacological activities. This prompted us to undertake the current comprehensive review, in which we covered different aspects related to *Z. lotus* including the botany, ethnobotany, phytochemistry, toxicology, geographical distribution, and pharmacology of this plant.

## 2. Methodology

In this comprehensive literature review, extensive bibliographic research was conducted to collect, analyze, and summarize the data on the botanical description, phytochemistry, ethnobotany, toxicology, and pharmacological activities of *Z. lotus*. Web of Science, Scopus, PubMed, ScienceDirect, SpringerLink, and Google Scholars were used to examine the published articles on *Z. lotus*. For this purpose, we used a list of keywords such as antioxidant activities, antidiabetic, antimicrobial effects, hepatoprotective effects, nephroprotective effects, analgesic effects, anticancer effects, anti-inflammatory effects, antiproliferative effects, and anti-obesity effects, combined with *Z. lotus*. The period in which we conducted the literature search was from 2021 to 2022. All English and French articles on *Z. lotus* were reviewed and included in this comprehensive review. The title of the investigation was used as the first step in identifying and reviewing the consolidated literature. If the study’s title and abstract were uncertain regarding the inclusion or exclusion from the current comprehensive study, the full text was examined. The chemical structures of the main phytochemical compounds present in the *Z. lotus* extracts were designed using ChemDraw 18.1 software.

## 3. Botanical Description

**Figure 1:**
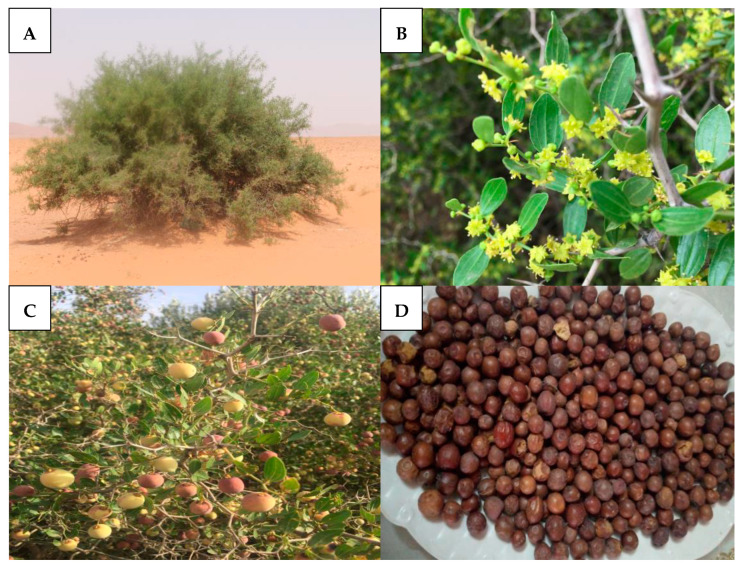
(**A**) Global view of *Z. lotus*. (**B**) Leaves of *Z. lotus* in the flowering stage. (**C**) *Z. lotus* fruits in ripening phase. (**D**) Mature *Z. lotus* fruits.

The *Ziziphus lotus* L. (*Z. lotus*) is a spiny fruit shrub, and a member of the family Rhamnaceae. It grows in tufts that are a few meters in diameter and up to two meters high, and is commonly called “Sedra” in North Africa (Figure 1A) [29]. Flowers of *Z. lotus* are small, axillary, bisexual, pentameric, and grouped in a cymose inflorescence, with sepals open in the star, small petals, and upper ovary, blooming in June–July (Figure 1B) [30]. The fruits of *Z. lotus* are drupes with welded stones, having the shape and size of a beautiful olive, first green, then young, and finally dark red at maturity from September to October (Figure 1C,D) [14]. The fructification begins in the fourth year in full yield around the age of fifteen; it is very productive when it receives copious watering during the summer [14]. The plant has oblong, whole-margined, short-petiolate, glabrous, alternating, deciduous leaves, and each leaf bears at its base two stipules transformed into an uneven and vulnerable thorn (Figure 1B) [14].

## 4. Eco-Geographical Features 

Typically, *Z. lotus* is found in dry and semi-arid regions, particularly in the Mediterranean region [16]. It grows in a few southern European nations including Greece, Italy, and Spain. It is also found in western Asia and is very common in northern Africa from Morocco to the Egyptian Sahara [31]. It reappears on the island of Socotra, in Yemen, and throughout the Middle East including Cyprus, Turkey, Palestine, and Syria [32]. It is also distributed in Iran, China, and South Korea [13]. *Z. lotus* can adapt to a wide range of climatic circumstances. Due to its late blooming, it is more resistant to winter frosts, up to −15 °C, than spring frosts (May–July) [11]. It is a sun plant reserved for warm and dry climates. It can withstand drought well and needs a lot of heat (maximum 45 °C) to bear fruit [31]. This species is adapted to areas with low rainfall [33]. A variety of soil types are tolerated by *Z. lotus*. It favors deep, sandy soils that are neutral to slightly alkaline and can withstand salt [31]. This plant can be encountered in desert areas with very low rainfall and grows in rocky areas, cliffs, and foothills. It reproduces vegetatively with a low propagation by seedling, its thermal optimum is 35 °C, and its germination is rare because the seedling requires the treatment of nuclei by the digestive juices of animals [34].

## 5. Ethnomedicinal Uses 

Table 1 summarizes the traditional knowledge on the therapeutic uses of *Z. lotus*. This plant is known in the North African region under the name “Sedra” and its fruits “Nbeg” [9,10,35]. Numerous ethnobotanical field works carried out in different regions of Morocco have revealed a broad range of traditional medicinal applications of *Z. lotus*. In fact, the leaves and seeds of this species are used in decoctions, raw or fresh, in the northeast of Morocco, to treat muscle ailments, diabetes, urinary tract problems, metabolic abnormalities, and skin and digestive problems [8]. However, in northeast Morocco, the leaves, roots, and fruits of this herb are used in powder, decocted, and in an infusion to treat kidney stones, renal colic, pyelonephritis, and polycystic kidney disease [36]. In the Moroccan region of Fez-Meknes, the fruits or leaves of *Z. lotus* are utilized against kidney stones [37]. In the High Central Atlas of Morocco, where the plant is known by its vernacular name “Azgour”, the fruit and the leaf in the decoction, infusion, and powder are frequently used as aperitifs, anti-ulcer remedies, anti-diabetic agents, and wound-healing agents [38]. In the Agadir Ida Outanane region, the decoction of seeds is used for problems with diabetes [39]. In Algeria, *Z. lotus* is commonly used for treating numerous health problems. Indeed, in the El-Bayadh region, the leaves in the decoction are used externally or internally as antitussive and antiseptic [40]. Additionally, the *Z. lotus* roots and leaves are extensively used in the Tlemcen region to cure diabetes, ulcers of the esophagus, colon, body aches, and arthritis [41,42]. The decoction, infusion, and powder of leaves and fruits are used in the M’Sila area of Algeria as an eczema treatment, anti-inflammatory, and pectoral support [43,44,45]. In the Algerian Sahara region (Oued Righ), the decoction and maceration of the parts of *Z. lotus* are used as tonics and diuretics, emollients, sedatives, and anti-inflammatories [46]. The study realized in the region of Ouargla indicated that the decoction and maceration of the fruits, leaves, and roots were used as moisturizers, sedatives, and diuretics [47]. In Mauritanian ancestral medicine, the traditional uses of *Z. lotus* are significantly broadened. In fact, the plant known as *“Sdar hreytek”* is strongly advised against stomach and epigastric pain in the district of Adrar in Mauritania [48]. In traditional Libyan medicine, the bark, fruit, leaves, and roots of *Z. lotus* are utilized against constipation, stomach disorders, hair parasites, gastritis, sciatica, abscesses, batteries, strengthening and activation, and hepatitis [49,50,51]. In Jordanian folk medicine, the seeds and fruits of the plant are recommended against cough, measles, deworming, and as an antispasmodic [52,53]. In Palestine, the plant is known under its local name “Zyzafun”, and its leaves are used as a disinfectant [54].

## 6. Phytoconstituents of *Z. lotus*

**Figure 2:**
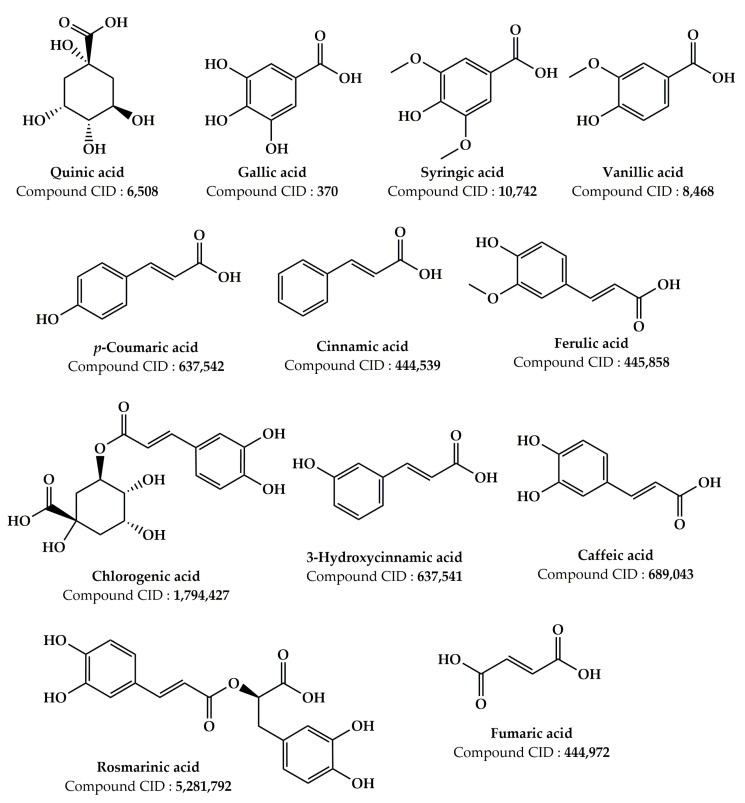
Chemical structures of the main phenolic compounds detected in the *Z. lotus* extracts.

**Figure 3:**
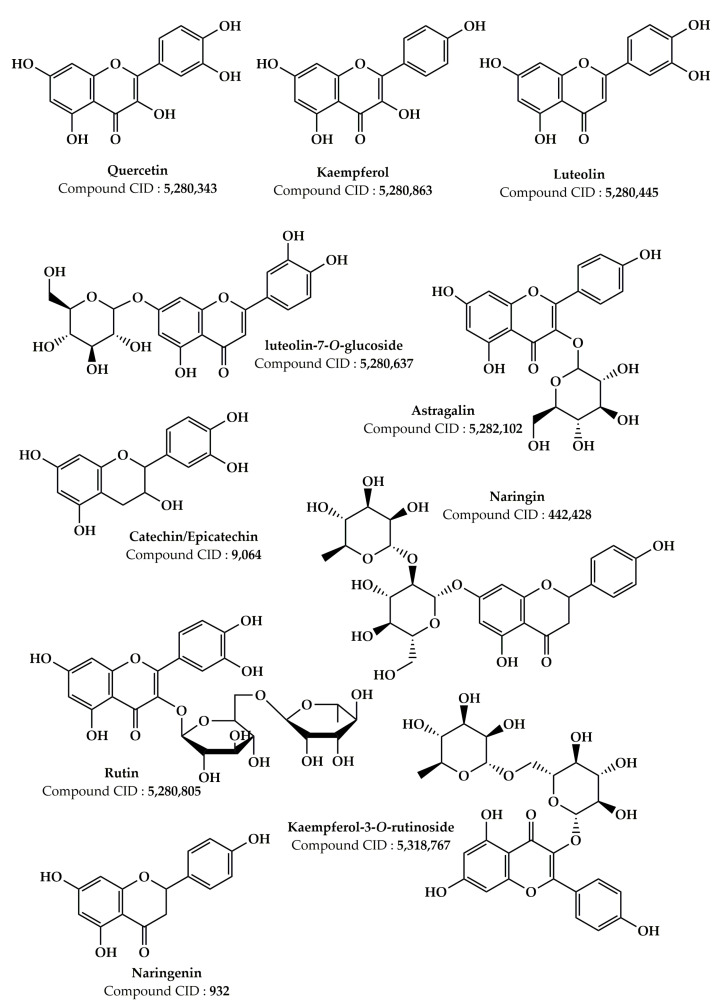
Chemical structures of the main flavonoid compounds found in the *Z. lotus* extracts.

**Figure 4:**
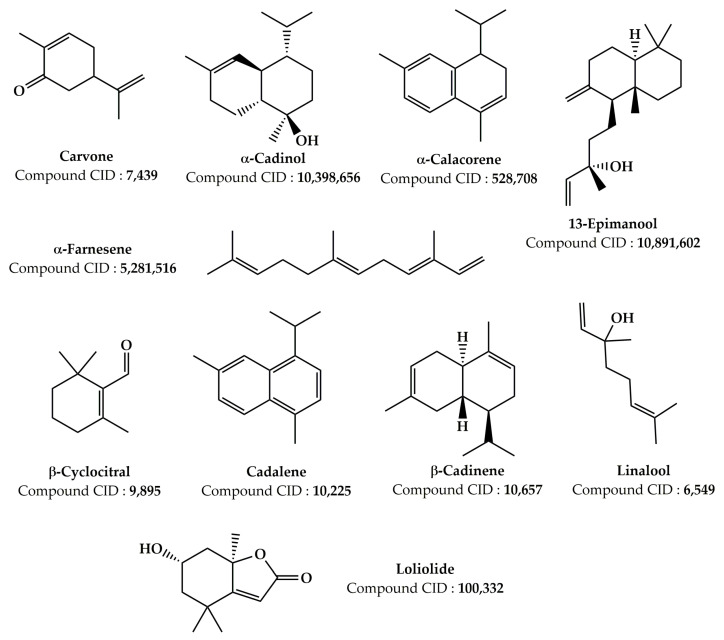
Chemical structures of the terpene compounds detected in the *Z. lotus* extracts.

**Figure 5:**
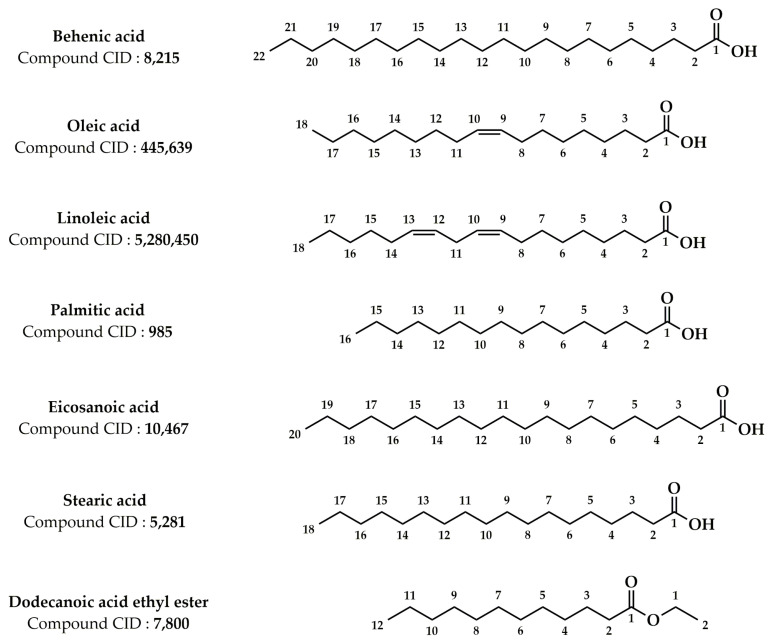
Chemical structures of the fatty acids identified in *Z. lotus*.

**Figure 6:**
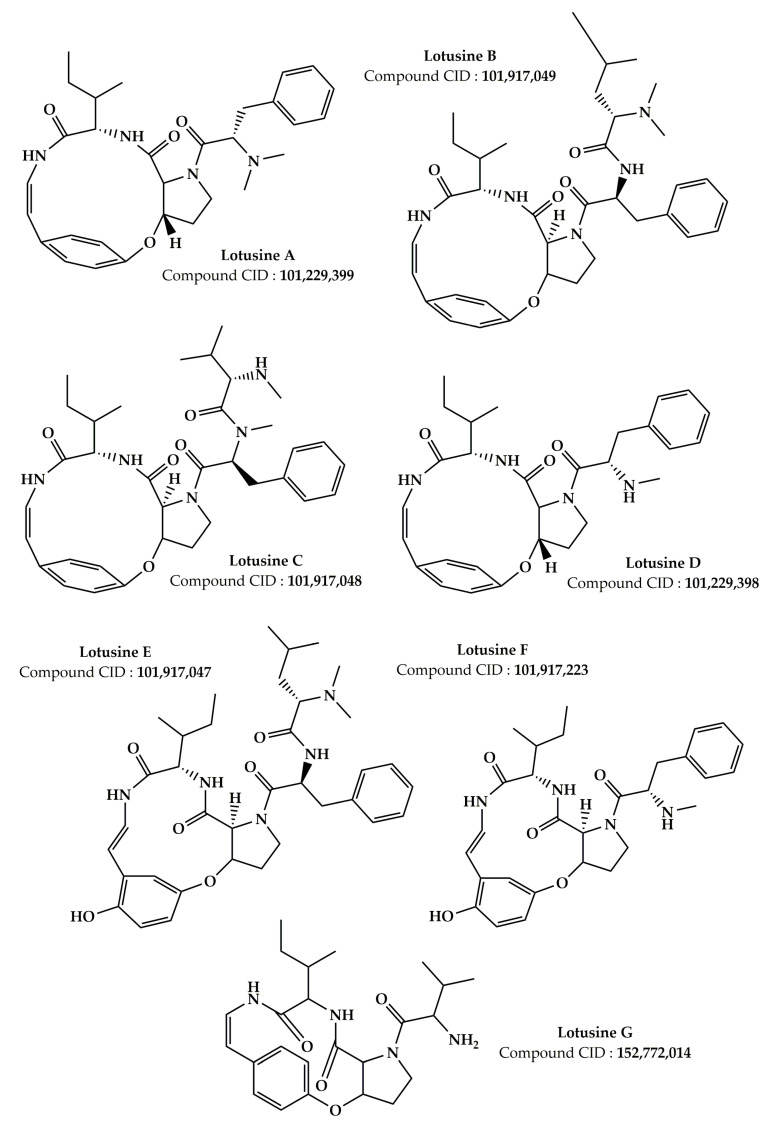
Chemical structures of the alkaloid compounds identified in *Z. lotus*.

Numerous earlier investigations focused on the phytochemical composition of extracts from various *Z. lotus* parts have been published [17,19,20,21,24,25,26,27,101,102,103,104,105,106,107]. Analysis of the data grouped in Table 2 allowed us to identify more than 181 phytochemical compounds present in the *Z. lotus* extracts from different countries (Tunisia, Morocco, and Algeria). These phytochemicals encompass a significant number of fatty acids and four significant families of secondary metabolites including phenolic acids, terpenoids, flavonoids, and alkaloids (Table 2). The chemical structures of the main phytoconstituents present in *Z. lotus* extracts are presented in the figures according to their chemical classes, phenolic acids (Figure 2), flavonoids (Figure 3), terpenes (Figure 4), fatty acids (Figure 5), and alkaloids (Figure 6). In fact, in the methanol extract of the leaves, seeds, and fruits of *Z. lotus* from Tunisia, 28 phenolic compounds were found by Yassine et al. (2020) using liquid chromatography-electrospray ionization-tandem mass spectrometry (LC-ESI–MS) analysis [19]. The authors of this study indicate that quinic acid, ferulic acid, epicatechin, rutin, quercitrin, naringin, and cinnamic acid are major phenolic compounds in this extract. In addition, 10 phenolic components including fumaric acid, catechin, tyrosol, gallic acid, syringic acid, *p*-coumaric acid, vanillin, ferulic acid, caffeic acid, and cinnamic acid were found in the methanol extract from Tunisian *Z. lotus* leaves using the high-performance liquid chromatography (HPLC) method [17]. Previous phytochemical studies have reported the identification of 15 different phytochemical compounds present in the methanolic extract of the fruits and leaves of Moroccan *Z. lotus*, namely galloyl shikimic acid, (-)-catechin 3-*O*-gallate, malic acid, quercetin, rhamnosyl-rhamnosylglucoside, quercetin di-glucoside, eriodictyol, macrocarpon C, amorfrutin A, isovitexin-2″-*O*-rhamnoside, hyperin, astragalin, 7,8-dihydrobiopterin, quercetin-3-galactoside, and kaempferol-3-diglucoside. According to other investigations, the aqueous extract of Moroccan *Z. lotus* fruits and leaves contains 22 distinct phenolic compounds including gallic acid, pyrogallol, chlorogenic acid, catechin, rutin, *p*-hydroxybenzoic acid, caffeic acid, vanillic acid, epicatechin, syringic acid, *p*-coumaric acid, 3-hydroxycinnamic acid, ferulic acid, sinapic acid, salicylic acid, rosmarinic acid, resveratrol, quercetin, naringin, catechol, hydroxytyrosol, and naringenin [20,21]. Other investigations have indicated that the aqueous extract of the branches and leaves of Algerian *Z. lotus* revealed the presence of 18 phenolic compounds, which were defined as quercetin-3-*O*-(2,6-di-*O*-rhamnosylglucoside)-7-*O*-rhamnoside, quercetin-3-*O*-(2,6-di-*O*-rhamnosylglucoside), myricetin-3-*O*-rutinoside, quercetin-3-*O*-(2,6-di-orhamnosylglucoside-7-*O*-glucuronide, kaempferol-3-*O*-(2,6-di-*O*-rhamnosylglucoside), phloretin-di-c-hexoside, quercetin-3-*O*-rutinoside, kaempferol-*O*-hexoside, kaempferol-3-*O*-(2,6-di-*O*-rhamnosylglucoside), oleuropein hexoside, kaempferol-3-*O*-rutinoside, kaempferol-3-*O*-(6-*O*-rhamnosyl-glucoside), apigenin-*O*-hexoside-*O*-deoxyhexoside, oleuropein, catechin, quercetin-*O*-deoxyhexoside, eriodictyol-*O*-deoxyhexoside, (Epi)catechin-(epi)gallocatechin, and (-)-epicatechin [26]. In addition, Ghedira et al. (1993, 1995) and Croueour et al. (2002) identified seven alkaloid compounds in the root bark extract of *Z. lotus*: lotusine A, lotusine D, lotusine B, lotusine C, lotusine E, lotusine F, and lotusine G [108,109,110].

Comparatively, we observed that the chemical composition of *Z. lotus* extracts varied from one country to another. This variation in the chemical composition between identical *Z. lotus* extracts from other countries may be provoked by a variety of elements including the collection’s origin, the climate during the harvest, the harvest season, and the extraction technique [111]. Letaief et al. (2021) studied the phytoconstituents of the essential oils from the aerial part of Tunisian *Z. lotus* using the gas chromatography-mass spectrometry (GC–MS) [107]. The authors of this investigation reported that the essential oil of *Z. lotus* contains 36 volatile compounds, with the major components being hexahydrofarnesyl acetone, followed by geranylacetone, cis-hexenyl-3-benzoate, 2-pentadecanone, dodecanoic acid ethyl ester, and *n*-hexadecanoic acid. In addition, Rais et al. (2020) showed the existence of fatty acids such as oleic acid, palmitic acid, stearic acid, and linoleic acid in the essential oil extracted from the seeds of Moroccan *Z. lotus* (Figure 3) [106]. Zazouli et al. (2022) studied the chemical composition of lipophilic fractions (dichloromethane extract) of different parts of the Moroccan *Z. lotus* using GC–MS (Table 2) [102]. A total of 99 lipophilic compounds including fatty acids, long-chain aliphatic alcohols, pentacyclic triterpenic chemicals, sterols, monoglycerides, aromatic substances, and other minor substances were identified and measured in this recent study. In reality, it has been shown that the majority of lipophilic extracts of the pulp, leaves, and seeds are composed of fatty acids. Unsaturated fatty acids, specifically acid (9Z,12Z)-9,12-octadecadienoic acid (9Z)-9-octadecaenoic, were notably abundant in the leaves and seeds. The root bark, on the other hand, was rich in pentacyclic triterpenic substances, particularly betulinic acid.

## 7. Pharmacological Activities

**Figure 7:**
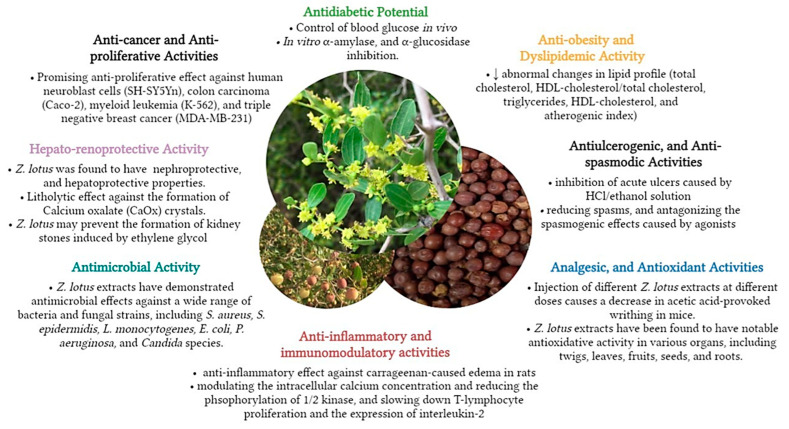
Comprehensive summary of the pharmacological activities of *Z. lotus*.

Modern pharmacological research has revealed in recent years that *Z. lotus* extracts contain a range of pharmacological features including anti-diabetic, anti-cancer, hepatoprotective, nephroprotective, anti-inflammatory, analgesic, anti-oxidant, and antibacterial actions. Numerous studies have revealed that the chemical constituents and raw extracts of *Z. lotus* have significant levels of biological properties. Figure 7 provides an overview of the mechanisms of the pharmacological activities demonstrated by *Z. lotus* extracts.

### 7.1. Antidiabetic Activity

Numerous diabetic patients have employed conventional herbal treatments in a variety of formulations as a supplemental therapy to manage problems with diabetes since Antiquity [112,113]. As indicated in Table 1, the *Z. lotus* plant is frequently used in traditional medicine to treat diabetes. The anti-diabetic activities of these plant extracts have been confirmed by several preclinical studies on animals. In fact, an in vivo investigation discovered that giving diabetic hamsters an aqueous extract of *Z. lotus* fruit at a concentration of 300 mg/kg controlled the blood glucose levels [114]. The authors of this study indicated that the anti-diabetic activity of the aqueous extract of *Z. lotus* fruit (300 mg/kg) was comparable to that of the drug glucophage 50 mg (metformin). In addition, the effect of the *Z. lotus* leaf and fruit extract on the inhibition of *α*-amylase and *α*-glycosidase was evaluated in vitro [20]. The finding of this survey indicates that the *Z. lotus* leaf and fruit extract has potent in vitro anti-diabetic effects via *α*-amylase inhibition (leaves: IC_50_ = 20.40 ± 1.30 μg/mL; fruits: IC_50_ = 31.91 ± 1.53 μg/mL), and *α*-glycosidase (leaves: IC_50_ = 8.66 ± 0.62 μg/mL; fruits: IC_50_ = 27.95 ± 2.45 μg/mL). The effect of this extract was stronger to that of the common acarbose prescription. The data gathered from this text indicate the anti-diabetic potential of *Z. lotus* extracts, and support their traditional uses as anti-diabetic drugs.

### 7.2. Anti-Obesity and Dyslipidemic Activity 

In recent years, hyperlipidemia has been considered as a major health illness. This factor is well-known to be the primary risk factor for the development of atherosclerosis as well as other related cardiovascular and brain vascular diseases [115]. Several previous investigations have been conducted to confirm the anti-hyperlipidemia activities of *Z. lotus* in the preclinical stage. Indeed, it has been demonstrated that the aqueous extract of *Z. lotus* fruits exerts anti-hyperlipidemic effects in albino mice fed a prolonged, fat-rich diet [35]. Results from this study indicate that the administration of the aqueous extract of *Z. lotus* fruit at 200 and 400 mg/kg for 30 days improved abnormal changes in the lipid profile (total cholesterol, HDL-cholesterol/total cholesterol, triglycerides, HDL-cholesterol, and atherogenic index) and blood glucose in albino mice subjected to a chronic high-fat diet. In addition, one study assessed the effects of *Z. lotus* fruits on diet-induced obesity in mice [116]. This study showed that taking 10% (*w*/*w*) of *Z. lotus* fruit powder supplemented with a high-fat diet for six weeks improved the plasma lipid concentrations, and thus the expression of key genes involved in energy metabolism and inflammation. In addition, an in vivo study demonstrated the anti-cholesterolemic effect of aqueous *Z. lotus* fruit extract in hamsters exposed to a high-fat diet [114]. Daily administration of an aqueous extract of *Z. lotus* fruit at a dose of 300 mg/kg in obese hamsters for 30 days substantially reduced the plasma level of bad cholesterol compared to obese animals not treated with the *Z. lotus* extract.

### 7.3. Antiulcerogenic and Anti-Spasmodic Activities

As indicated in Table 1, *Z. lotus* is utilized in herbal medicine in several countries to treat digestive tract pathologies such as constipation, diarrhea, and spasms. Several previous preclinical studies have shown the beneficial effects of *Z. lotus* extracts against some digestive problems. Bakhtaoui et al. (2014) showed that the administration of *Z. lotus* fruit methanol extract at 500 mg/kg had anti-ulcerogenic effects provoked by HCl/ethanol, pyloric ligature, and aspirin in Wistar rats [117]. Another study found that the oral administration of aqueous extracts of *Z. lotus* root bark, leaves, and fruits resulted in a substantial and dose-dependent inhibition of acute ulcers caused by the HCl/ethanol solution [118]. According to the same authors, this effect of *Z. lotus* extracts is comparable to that of cimetidine and omeprazole (standard drugs). Furthermore, the antispasmodic effects of aqueous and methanol extracts of *Z. lotus* leaves and root bark were tested on male rats [119]. The results of this investigation showed that *Z. lotus* root bark and leaf extracts were able to relax the tone of spontaneous duodenum contractions in rats and antagonize the spasmogenic effects caused by agonists such as acetylcholine, KCl, and BaCl_2_. The authors explain this effect by the fact that the extracts of *Z. lotus* clumped on the cholinergic receptors and blocked the influx of Ca^2+^.

### 7.4. Anti-Inflammatory and Immunomodulatory Activities

Inflammation is typically characterized as a reaction to an injury or infection [120]. It is now widely recognized that chronic inflammation is always linked to diseases of wealth and prolonged longevity including cancer, obesity, cardiovascular, and neurological disorders [120,121,122,123]. Inflammation is characterized by four primary symptoms: redness, heat, swelling, and pain. The body naturally responds to damaging stimuli by inducing inflammation, which is accomplished by the migration of plasma and leukocytes into damaged tissues. This particular immune response, which is categorized as acute inflammation, is crucial for the body to fend off dangerous microorganisms [124]. It is critical to identify chemicals that can aid in the resolution of inflammation in this situation in a way that is homeostatic, modulatory, effective, and well-tolerated by the body. In this context, a study revealed that *Z. lotus* extracts had an anti-inflammatory effect against carrageenan-caused edema in rats [29]. According to the findings of the study, administering an aqueous extract of the *Z. lotus* root bark intraperitoneally at doses of 50, 100, and 200 mg/kg significantly reduced the paw swelling caused by carrageenan three hours later by 37.81%, 69.18%, and 72.90%, respectively. Additionally, a considerable activity occurred at a dose of 200 mg/kg, three hours after the injection of carrageenan, after taking the methanolic extract, with a reduction of 67.57% in the volume of the paw. The authors of this study showed that this anti-inflammatory effect was close to that of the standard drugs (piroxicam). Furthermore, previous research indicates that extracts from various parts of *Z. lotus* have immunosuppressive properties. The methanolic extract of *Z. lotus* has the important property of modulating the changes in intracellular calcium concentrations caused by thapsigargine in human T Jurkat lymphocytes. It also has the ability to reduce the phosphorylation of the 1/2 kinase controlled by the extracellular signal (ERK1/2) as well as the proliferation of T-lymphocytes by slowing their progression from the S phase to the G2/M phase of the cell cycle and the expression of interleukin-2 (IL-2) [16]. Similarly, the aqueous extract of *Z. lotus* pulp, seeds, leaves, roots, and stems had a significant immunosuppressive effect, inhibiting the T lymphocyte proliferation [125].

### 7.5. Analgesic Activity

As a sensory modality, pain frequently serves as the sole indicator for the diagnosis of a number of disorders. It frequently serves a defensive purpose. Humans have employed a variety of therapies throughout history to relieve pain, with medicinal herbs standing out due to their widespread use [126]. In this regard, Borgi et al. (2007) reported that *Z. lotus* extracts have an analgesic activity in the preclinical stage [29]. In this investigation, it was shown that intraperitoneal injection of the aqueous, methanol, chloroform, and ethyl acetate extracts at doses of 50, 100, and 200 mg/kg caused a decrease in acetic acid-provoked writhing in mice. The same authors claimed that the analgesic effects of the ethyl acetate extract were extremely effective in comparison to the other tested extracts.

### 7.6. Anti-Cancer and Anti-Proliferative Activities

Cancer establishes when cells divide rapidly and invade the surrounding tissue before spreading to other parts of the body [127]. The key aim of anticancer therapy is to cure the illness while also attempting to extend and boost the quality of a patient’s condition [128]. Since chemotherapy has harmful effects on cancer treatment, alternative therapies based on bioactive cytotoxic substances are an important help in cancer prevention. Preclinical studies conducted using methanolic, aqueous, petroleum ether, dichloromethane, and acetonic extracts of *Z. lotus* have revealed the potential mechanisms of anticancer activity. Letaief et al. (2021) evaluated the cytotoxic activity of petroleum ether and dichloromethane extract from *Z. lotus* roots against the SH-SY5Yn cell line using the MTT assay [25]. The findings of this study indicate that all of these human neuroblast cells are very sensitive to extracts of petroleum ether and dichloromethane of the *Z. lotus* roots, with varying IC_50_ values after 24 h of treatment (IC_50_ = 184.413 ± 4.77 µg/mL), after 48 h of treatment (IC_50_ = 20.941 ± 1.16 µg/mL), and after 24 h of treatment (IC_50_ = 16.148 ± 0.93 µg/mL, and after 48 h of treatment (IC_50_ = 7.341 ± 1.98 µg/mL), respectively. Furthermore, Tlili et al. (2019) examined the cytotoxicity of an ethanolic extract of the aerial part of *Z. lotus* against two human cancer cell lines, colon carcinoma (CaCo-2) and myeloid leukemia (K-562) [104]. The results of this study showed that this extract inhibited the proliferation of CaCo-2 and K-562 with an IC_50_ below 50 μg/mL. Moreover, the *Z. lotus* root bark lipophilic extract displayed promising antiproliferative effects against MDA-MB-231, a triple negative breast cancer cell line, with an IC_50_ = 4.23 ± 0.18 µg/mL [102].

### 7.7. Hepato-Renoprotective Effects

Bencheikh et al. (2021), investigated the nephroprotective efficacy of an aqueous extract of *Z. lotus* fruits [21]. The authors utilized gentamicin (GM), an antibiotic aminoglycoside used to treat severe acute illnesses, to produce nephrotoxicity in rats, and examined the preventative efficacy of the aqueous extract of *Z. lotus* fruits as a result. GM caused a significant biochemical imbalance (an increase in blood urea, creatinine, and uric acid as well as a decrease in urine) and significant deterioration in renal function was evident in this imbalance. However, daily administration of the aqueous extract from *Z. lotus* fruits three hours prior to GM injection successfully restored these GM-induced defects. The plant extract’s efficacy was dose-dependent, with the highest effect reported at 400 mg/kg. These findings suggest the use of *Z. lotus* fruits as a nephroprotective agent due to their activities in improving the altered parameters during a nephrotoxicity condition [21]. In addition, Bencheikh et al. (2019) reported that *Z. lotus* fruits had hepatoprotective properties [22]. The authors utilized carbon tetrachloride (CCl_4_) as a toxic agent in this investigation to induce oxidative stress and hepatotoxicity in rats. This treatment led to a significant decrease (*p* < 0.001) in body weight as well as a large rise (*p* < 0.001) in the relative liver weight. Furthermore, the activity of plasma liver indicators (AST, ALT, and ALP) rose considerably (*p* < 0.001). It also had the ability to dramatically increase (*p* < 0.001) the concentration of direct and total plasma bilirubin as well as the levels of triglycerides (*p* < 0.05) among other liver biomarkers. Moreover, the authors assessed indicators of renal excretory function by assessing the plasma levels of creatinine, urea, and uric acid. Interestingly, the addition of the aqueous extract of *Z. lotus* fruits demonstrated substantial protection against CCl_4_-induced hepatotoxicity and nephrotoxicity. Indeed, the aqueous extract *Z. lotus* fruits restored almost all serum indicator enzymes and antioxidant status, bringing all values back to normal, indicating that the extract’s protective action in rats was mediated by reducing oxidative damage and liver injury [22]. The litholytic activity of the aqueous extracts of *Z. lotus* fruits and leaves was assessed in vitro and in silico [101]. Furthermore, the aqueous extract of *Z. lotus* fruits and leaves was found to suppress the production of CaOx crystals, induced by the addition of 0.1 mol/L oxalic acid in human urine, to form calcium oxalate (CaOx) crystals. The existence of bioactive chemicals detected by HPLC such as adenosine, isorhamnetin 3-*O*-rutinoside, *p*-hydroxybenzoic acid, and neoechinulin A was demonstrated by this litholytic action. In silico tests revealed that the discovered compounds work by targeting enzymes involved in calcium control, urate management, and acid–base homeostasis maintenance as well as having anti-inflammatory characteristics [101]. A study by Kouchalaa et al. (2017) evaluated the litholytic effects of the aqueous extract of *Z. lotus* on the dissolution of calcium oxalate and uric acid calculations in vitro [129]. The findings showed that at the end of the experiment, the ability of the aqueous extract to dissolve the calcium oxalate calculation was 7.65%, while the dissolution of the uric acid calculation was 10.75%. The high quantities of polyphenols and flavonoids in the extract were linked to the findings, according to the authors. Chakit et al. (2022) demonstrated that the aqueous extract of *Z. lotus* fruit had an anti-urolithic action in rats induced by ethylene glycol [130]. In the same ethylene glycol-induced urolithiatic model of rats, the administration of the aqueous extract of *Z. lotus* fruits dramatically decreased and prevented the formation of kidney stones and significantly alleviated renal impairment. The observed findings may be attributed to the tendency for urine alkalinization in *Z. lotus* fruit aqueous extract-treated mice is likely to have a role in oxalate crystal solubilization, which might potentially be one of the processes via which plant components act [130]. Calcium oxalate (CaOx) crystals were formed after the addition of 0.1 mol/L oxalic acid. The effect of aqueous extracts was compared to two reference antagonists (citrate and magnesium).

### 7.8. Antimicrobial Activity

Worldwide, bacterial diseases account for a significant portion of mortality and morbidity. Inappropriate and excessive use of antibiotics has resulted in the development of resistance, which is making treatment more difficult as antibiotic resistance rises [131]. Therefore, it has become more crucial than ever to create novel antibiotics that can withstand the array of bacterial resistance mechanisms. To this end, the intention has been focused in recent years on the search for natural-based new therapeutic agents, particularly medicinal plants. The latter could be a viable area for researching the capacity of natural antimicrobials to suppress and/or destroy bacteria [132]. In this direction, *Z. lotus* extracts have demonstrated antimicrobial effects against a wide range of bacteria, namely, *Bacillus pumilus*, *Enterococcus faecalis*, *Listeria monocytogenes*, *Micrococcus luteus*, *Rhizobium* sp., *Staphylococcus aureus*, *Staphylococcus epidermidis*, *Agrobacterium* sp., *E. coli*, *Helicobacter pylori*, *Pseudomonas aeruginosa*, and *Salmonella Typhimurium* and also show effects against two fungal strains (e.g., *Candida albicans* and *Candida tropicalis*). 

Table 3 summarizes the antimicrobial properties of the *Z. lotus* extracts. Evidently, the acetonic extract of *Z. lotus* leaves had significant antibacterial activity against *S. aureus*, *S. aureus* methicillin-resistant, *S. epidermidis*, *S. epidermidis* methicillin-resistant, and *L. monocytogenes*, with the MIC ranging from 250 to 1000 µg/mL and MBC ranging from 500 to 2000 µg/mL [105]. Another study revealed that lipophilic extracts of various parts of *Z. lotus* have antibacterial potential against *E. coli*, *S. aureus*, and *S. epidermidis* strains, with MICs ranging from 1024 to 2048 µg/mL [102]. At a concentration of 10 mg/mL, the methanolic extract of *Z. lotus* leaves inhibits the growth of *S. aureus, L. monocytogenes, S. typhimurium,* and *E. coli*, with inhibition zones ranging from 10 to 13 mm [19]. Furthermore, the results of a study on ethanolic, methanolic, and aqueous extracts of *Z. lotus* seeds showed that these extracts have significant antibacterial activity against *E. coli, P. aeruginosa, S. aureus,* and *E. faecalis* with MICs ranging from 50 to 200 mg/mL [18]. Thus, the antimicrobial activity of the methanolic extract of *Z. lotus* stems was evaluated using the agar-well diffusion method against pathogenic microbial species *S. aureus, E. coli,* and *P. aeruginosa* [133]. The results of this study indicate a potential antibacterial activity of this extract with MIC values ranging from 6 to 7 mg/mL. In addition, the study of the antibacterial activity of the methanolic extract of *Z. lotus* fruits through the disc diffusion and micro-dilution method showed that this extract had an interesting activity against *E. coli*, *Agrobacterium* sp., *Rhizobium* sp., *B. pumilus*, and *B. subtilis* with a MIC range from 3.2 to 400 µg/mL [134]. Growth inhibition activities of *Z. lotus* leaf methanol extract against bacterial species, notably *B. subtilis, S. aureus, E. coli, P. aeruginosa, S. Typhimurium*, were assessed using the conventional paper disk test [135]. The findings of this study indicate that the *Z. lotus* leaf methanol extract inhibited bacterial growth with a MIC in the ranges of 12.5 to 1000 μg/mL.

### 7.9. Antioxidant Activity

An imbalance between the quantity of reactive oxygen species and free radicals, which have unpleasant side effects, and the body’s natural anti-oxidative defense mechanisms, results in oxidative stress [137,138]. In recent decades, researchers have concentrated on discovering naturally occurring anti-oxidizing chemicals that can counteract the potentially dangerous effects of free radicals [139]. In this respect, studies on the ability of *Z. lotus* extracts to bind free radicals have revealed that the various organs (twigs, leaves, fruits, seeds, and roots) have a notable anti-oxidative activity. The anti-oxidative capacity tests were performed using the DPPH, FRAP, TAC, *β*-carotene bleaching assay, and ABTS methods. The following table (Table 4) summarizes the results obtained in various countries throughout the world. Several studies have been conducted to assess the anti-oxidative potential of extracts from different parts of *Z. lotus* in Morocco, Algeria, Tunisia, and Italy. In Morocco, the anti-oxidant ability of the aqueous extract of *Z. lotus* fruits was found to be interesting with IC_50_ = 116 ± 0.02 µg/mL, and an important inhibition of *β*-carotene oxidation, 21.11% at 100 µg/mL, tested by the mean of DPPH and the *β*-carotene assays, respectively [35]. Aya et al. (2020) highlighted that the various extracts (namely, hexane, methanol, and dichloromethane extracts) of the fruits and the leaves of *Z. lotus* possessed an interesting DPPH radical scavenging ability, ranging from an IC_50_ equal to 0.70 mg/mL, and an IC_50_ >40 mg/mL, with the methanolic extract of *Z. lotus* leaves being the most potent antioxidant (IC_50_ = 0.70 mg/mL) among the other tested extracts [101]. Another report found that the methanolic, and ethanolic extracts from *Z. lotus* seeds expressed an anti-oxidant activity, corresponding to the IC_50_ = 1.33 ± 0.01 mg/mL, and IC_50_ = 1.32 ± 0.09 mg/mL, respectively [18]. A comparative study of the anti-oxidant abilities of the aqueous extract of *Z. lotus* fruits and leaves was conducted by [20] by the mean of three anti-oxidant methods (DPPH, ABTS, and FRAP). In this sense, the radical scavenging potential of the aqueous extract of the fruits was higher than that of the leaves in the three anti-oxidant assays, as shown in Table 4. The observed results were attributed to the high level of phenolics and flavonoid compounds in the fruits. Other Moroccan, Tunisian, Algerian, and Italian research teams have confirmed that the radical-scavenging ability is more important in *Z. lotus* fruits, regardless of the nature of the solvent. In this context, the crude methanol extract of *Z. lotus* fruits from Oued Esseder (southeastern Tunisia) showed an important scavenging effect against DPPH radicals with an IC_50_ = 15.15 ± 0.90 µg/mL as well as an important total antioxidant capacity of 25.02 ± 0.55 mg GAE/g EDW. The same results were obtained for the methanolic extracts of the fruits from Bengardane (southeastern Tunisia) [19]. Ait Abderrahim et al. (2017) examined the anti-oxidant capacity of the methanolic extract of *Z. lotus* stems in vitro using DPPH [133] and reported a remarkable anti-oxidant result of 480.20 ± 40.64 mg AAE/g EDW. An Italian team investigated the antioxidant effect of the methanolic extract of stem bark from *Z. lotus* (from Addaura, Palermo, Italy) using three different methods [140]. They recorded a strong capacity to chelate ferrous ions from the ferrozine complex (39.01 ± 4.30 mg ethylenediaminetetraacetic acid equivalents, EDTA/g extract). The tested extract exhibited a potent DPPH free radical-scavenging effect displayed by the equivalent of ascorbic acid (304.02 ± 4.80 mg AAE/g EDW). For the FRAP test, a strong lowering power in the FRAP test was observed (296.68 ± 1.81 mg TE/g EDW) [140].

### 7.10. Others Activities

Human skin enzymatic browning entails the production of melanin, which proceeds through numerous steps involving multiple enzymes [144]. Within these enzymes, tyrosinase is implicated in the first two steps, and its inhibition could be used to build skin-protective medicines. Inhibiting the tyrosinase activity is an essential skin protection approach. The dermatoprotective activity of the extracts of *Z. lotus* fruit and leaves was investigated in the work of Marmouzi et al. (2019) [20]. The aqueous extract of the *Z. lotus* fruits demonstrated a higher tyrosinase inhibitory activity than the leaf extract, with IC_50_ values of 70.23 ± 5.94 μg/mL and 129.11 ± 9.40 μg/mL, respectively. Both extracts were more effective than the reference substance utilized (quercetin) with 246.90 ± 1.90 µg/mL. The difference in inhibitory activities between the *Z. lotus* fruit and leaf extracts is mostly related to chemical functional component differences. The extract of *Z. lotus* fruits is high in phenolic components such as catechin, gallic acid, and rutin. These substances are recognized tyrosinase inhibitors [144]. 

Khazri et al. (2017) evaluated the neuroprotective effect of the *Z. lotus* fruit extract against the neurotoxicity-induced by cypermethrin (CYP), a synthesized pyrethroid employed as an insecticide in large-scale agricultural applications [145]. CYP administration in mice resulted in a substantial increase (*p* < 0.05) in the heart, liver, and kidney oxidative markers (H_2_O_2_ and catalase) as well as an increase (*p* < 0.001) in the MDA levels in the heart, liver, and kidney. The current study also found that once 150 µg/L of CYP administered to mice significantly inhibited the AChE activity, when referred to healthy mice, this decrease (*p* < 0.05) in AChE activity was observed in all organs. The administration of the *Z. lotus* fruit extract successfully preserved standard biochemical parameters in mice against the toxicity generated by CYP. These findings support the pharmacological efficacy of the *Z. lotus* fruit extract in CYP-induced oxidative stress, suggesting the use of the extract as a neuroprotective agent

## 8. Toxicology

The plant’s security was proposed by its widespread use as a food and in ethnomedicine for a range of ailments, with no mentioned harmful effects. Bencheikh et al. (2019) investigated the acute oral toxicity of the aqueous extract of *Z. lotus* fruit in mice. After 14 days of observation, they found that a single oral dosage of this extract at 2000 mg/kg body weight did not cause any animal fatalities or changes in animal behavior [22]. Bekkar et al. (2021) found that a single dose of 5000 mg/kg body weight did not exhibit a toxic effect, death, or change in behavior after 14 days [23]. According to the findings of this plant’s acute toxicity, an acute use of this plant could be safe. To validate the safety of the long-term application of *Z. lotus* species, more studies on sub-chronic and chronic toxicity assessment should be carried out.

## 9. Concluding Remarks and Future Prospects

We emphasized the studies on the ethnobotanical, phytochemical, toxicological, and pharmacological properties of *Z. lotus* in this review. Several countries, especially those in North Africa, employ this plant extensively in herbal medicine to cure a broad range of illnesses including diabetes, digestive system issues, urinary tract issues infectious diseases, cardiovascular disorders, neurological diseases, skin issues, and others. According to the most recent pertinent data, several bioactive substances have been identified and isolated from *Z. lotus* extracts. These chemicals are secondary metabolites that belong to the flavonoids, phenolic acids, terpenoids, alkaloids, and other classes. In various scientific studies, the pharmacological assessment of *Z. lotus* revealed interesting medicinal activities. Indeed, *Z. lotus* fruit was observed to be the most efficient component of the plant in terms of treating diabetes, obesity, dyslipidemia, ulcers, and spasms. Additionally, it was found to have an anti-urolithic effect, preventing the formation of kidney stones. Furthermore, *Z. lotus* fruits showed significant protective effects against both CCl_4_-induced hepatotoxicity and GM-induced nephrotoxicity in rats. It was found that the fruit extract of *Z. lotus* demonstrated a higher inhibitory activity against tyrosinase compared to the leaf extract. For the neuroprotective effect of the plant against neurotoxicity induced by CYP, the *Z. lotus* fruit extract helped preserve standard biochemical parameters in mice against the toxicity generated by CYP, suggesting the potential use of the extract as a neuroprotective agent. The fruits and leaves also exhibited the ability to inhibit the production of CaOx crystals, induced by the addition of oxalic acid in humans. Research has demonstrated that the root bark of *Z. lotus* possesses properties that can help to reduce inflammation, and has immunosuppressive abilities as it can regulate intracellular calcium levels and reduce T-lymphocyte proliferation. Regarding the antimicrobial activity, all parts of the plant including the leaves, stems, seeds, and fruits displayed noteworthy antibacterial activity against a variety of bacterial strains including *S. aureus*, *E. coli*, and *P. aeruginosa*. However, the efficacy of each part may vary depending on the specific bacteria and extraction method used. Several studies have shown that different parts of the *Z. lotus* plant such as twigs, leaves, fruits, seeds, and roots possess significant antioxidant properties. The antioxidant capacity of these extracts was tested using various methods such as DPPH, FRAP, TAC, *β*-carotene bleaching assay, and ABTS, which demonstrated varying levels of antioxidant activity. These in vivo and in vitro pharmacological confirmations confirm the traditional uses of *Z. lotus* extracts. However, the studies on the pharmacological properties discussed in this paper did not show the underlying pathways by which *Z. lotus* extracts act. Using data from the literature, some studies suggest a positive relationship between pharmacological properties and some of the isolated *Z. lotus* compounds in some bioactivities. To fully understand the mechanism of action of the bioactive compounds found in *Z. lotus*, additional study on this topic is necessary. The toxicological testing of *Z. lotus* extracts on animal models revealed no significant acute toxicity. However, further research is required to assess the toxicity at various dosages and over various time periods. Clinical investigations are urgently required to support the usage of this herb since controlled trials were not performed. 

## Figures and Tables

**Table 1:** Ethnobotanical uses of *Z. lotus*.

Country	Region	Vernacular Name	Parts Used	Mode of Preparation	Mode of Administration	Therapeutic Uses	Reference
Morocco	Northeastern	Asadra, Nbeg, Tazakort	Leaves, seeds	Decoction, raw, or fresh	Oral	Digestive problems, skin problems, nervous system disorders, diabetes, urinary tract problems, endocrine and metabolic disorders, and muscles diseases	[8]
	Northeastern Morocco including eight province districts	-	Flowers, leaves, roots	Decoction, infusion, powder	-	Diabetes, urinary infections, antispasmodic, kidney diseases, hair care, circulatory disorders, and respiratory problems	[9]
	Region of Fez-Meknes	Nbeg	Fruits, leaves	Decoction	-	Kidney stones	[37]
	High Atlas Central Morocco	Ssedra, Azgour	Fruits, leaves	Decoction powder	-	Antiulcer, antidiarrheal, anorexia	[55]
	High Atlas Central of Morocco	Ssedra, Azgour	Fruits, leaves	Decoction, infusion, powder	-	Antidiarrheal, promotes the healing of wounds, antiulcer, aperitif, antidiabetic,	[38]
	High Atlas Central of Morocco	Ssedra, Azougar	Fruits, leaves, roots	Decoction, infusion, powder	-	Diabetes	[55]
	Middle Oum Rbia	Sdar, Nbeg	Fruits, leaves, seeds	-	-	Digestive, dermatological, genitourinary, cardiovascular, metabolic	[56]
	Guercif Province	Sadra	Roots	Maceration	-	Diabetes, intestinal pain	[57]
	Northeastern Morocco	Asadra, Nbeg	Leaves, fruits, roots	Decoction, infusion, powder	Oral	Urine retention, diuretic, renal colic, pyelonephritis, polycystic kidney disease, and kidney stones	[36]
	Markets of Salé Prefecture, Northwestern Morocco	Sedr	-	Decoction	-	COVID-19	[58]
	Rif, Northern Morocco	Nbeg, Tazart	Seeds	-	-	Digestive system disorders	[59]
	Region of Tadla Azilal	Nbeg	Fruits, leaves	Powder	-	Gastrointestinal disorder	[60]
	Rabat-Sale-Kenitr	Nbeg	Fruits, leaves	Decoction	-	Chronic kidney diseases	[61]
	Taza	Sadra	Fruits, leaves	Infusion, powder	Externally, oral	Kidney problems, digestive system, diabetes, antimicrobial, hair care	[62]
	Moulay Yacoub Region	Asadra	Fruits	Infusion, powder	Oral	Stomach ache, hair care	[63]
	Tarfaya Province	Seder	Leaves	Powder	Oral	Kidney stones	[64]
	Province of Tarfaya	Ssder	Fruits, leaves	Powder, poultice	Oral, externally	Kidney stones, stomach pain, hair loss	[65]
	Province of Sidi Kacem	Ssedra	Seeds	Raw	-	Digestive infection	[66]
	Region of Fez-Meknes	Sidra, Nbeg	Seeds	Decoction,	-	Acute ache, digestion problems, intestinal comfort, bloating	[67]
	Er-Rich region	Azouggar	Fruits	Powder	-	Stomach pain, colon pain, anemia	[68]
	Nador Province	Thazagorth, Sidra	Fruits, leaves	Decoction; powder	-	Digestive diseases, diabetes	[69]
	Northeastern of Morocco	Sedra	Leaves, stems	Infusion	Oral	Headache, joint pain	[70]
	Province of Taroudant	Azougar, Sedr, Nbeg	Roots	Infusion	Oral	Diabetes	[71]
	Northeastern Morocco	Sedr, Nbeg	Roots	-	-	Digestive disease, diabetes	[72]
	-	Nbague	Fruits	Decoction, infusion	Oral	Kidney stones	[73]
	Region of Fez-Meknes	Sidra, Nbeg	Seeds	Decoction		Diabetes, kidney problems	[74]
	Taza Region	Nbeg	Leaves	Decoction, powder	-	Diabetes	[75]
	Agadir Ida Outanane region	Azegar, Sedra, Nbeg	Seeds	Decoction	-	Diabetes	[39]
Algeria	El-Bayadh	Sedra	Leaves	Decoction	Externally use, internally use	Antitussive, antiseptic	[40]
	Region of Ouargla	-	Fruits, leaves, roots	Decoction, maceration	-	Anti-inflammatory, moisturizer, sedative, diuretic	[47]
	Tlemcen	-	Roots	Decoction	Oral	Diabetes	[41]
	The region of Hodna (M’Sila)	Sedra	Leaves	Infusion	-	Eczema	[44]
	North and southwestern Algeria	Sadra	Leaves	Decoction	-	Diabetes mellitus	[76]
	Region of Tiaret	Sedra	-	-	-	Pulmonary affections	[77]
	M’Sila (North Algeria)	Sedra	Leaves	Decoction, infusion, powder	-	Anti-inflammatory, wound-healing, dermal eczema	[78]
	Djebel Messaad Region (M’Sila, Algeria)	-	Fruits, leaves, roots	Decoction	-	Anti-inflammatory, emollient, pectoral	[43]
	M’Sila	Sedra	Leaves	Bath, infusion, lotion	-	Hair loss	[79]
	Djebel Zdimm (Setif)	-	Fruits, leaves, seeds	-	-	Stomach acidity, hypertension	[80]
	Hoggar, Algeria	Tabakat	Fruits, leaves	Decoction, powder	-	Digestive diseases, diarrhea, diabetes	[81]
	Southeast of the capital of Hodna (Algeria)	Sedra	Leaves	Lotion	-	Fever, eye diseases	[82]
	Oued Righ (Algerian Sahara)	Nbak, Sedra	Fruits, leaves, roots	Decoction, maceration	-	Tonic, emollient, sedative, anti-inflammatory, pectoral, diuretic	[46]
			Roots	Decoction	-	Gastrointestinal tract diseases, liver diseases	
			Fruits	-	-	Respiratory system	
	Adrar and Bechar		Roots	Infusion	Oral	Diabetes	[83]
			Fruits	Decoction, raw	-	Renal disorders	
			-	Decoction	-	Infections	
				Raw	-	Hair loss	
	West of Bordj Bou Arreridj (El Mansourah), Algeria	Sedra	Fruits, roots	Powder, raw	-	Pectoral, emollient activity, hepatic, chlorosis, lungs diseases, jaundice	[84]
	Algerian Semi-Arid Region	E’ssedra	Fruits, leaves, roots	-	-	Measles, constipation, diuretic, hair care, heart diseases, hyperglycemia, renal pain, renal stones, stomach ache, urinary infections	[85]
	East of Algeria	Essedra	Leaves	Infusion	-	Skin, digestive	[86]
	Region of Bissa	Sedra	Roots	Decoction	White washing	Toothache	[87]
	Northeast	Sedra	Roots	Powder, raw	-	Pulmonary affections, jaundice	[88]
	Northeastern Algeria	Sedra	Aerial parts, roots	Infusion	-	Diabetes	[89]
	Region of Aures	Thazzgarth	Fruits	Decoction used in mixture with honey, and Algerian tea	-	Urinary calculus	[90]
	Southeast of M’Sila (rural communities of Ben Srour)	Sadra	Fruits, leaves, roots	Cataplasm, powder, raw	-	Pulmonary affections, jaundice, eczema, emollient, stomach pain, headache	[91]
	Tlemcen National Park (extreme northwest of Algeria)	Essadra	Leaves, roots	Decoction	Oral	Stomach ache, colon, body pain, arthritis	[42]
	El Hammadia region	Sedra	-	-	-	Lungs diseases, jaundice, emollient	[91]
	El Kantara	Sedra, Nebag	Fruits, leaves	Chewing, decoction	-	Heartburn, constipation, weakness of heart, diuretic, breastfeeding	[92]
	Northwestern of Algeria	Sedra	Leaves	Powder with honey	-	Breast cancer	[93]
	Algerian Central Steppe Region (Djelfa)	Sedra	Leaves, roots	Decoction, lotion, maceration	-	Insomnia, renal diseases, hydatid cysts	[94]
	Region of Tiaret, Northwest of Algeria	Sedra	Fruits, leaves, roots	Decoction, infusion	Oral	All reported ailments	[95]
Mauritania	Adrar Province	Sdar hreytek	Aerial parts, fruits	Chewing, infusion, powder	Oral	Abdominal pain, epigastric	[48]
			Leaves	Macerated white water		Fever	
				Powder, maceration	Oral	Hypertension	
				Infusion	Oral	Non-insulin dependent diabetes	
			Fruits	Powder	Oral	Kidney symptoms	
Libya	Al-Jabal Al-Akhder, Wadi Alkuf, Libya	Sidr, Nabq	-	-	-	Hair parasites, sciatica, abscess, piles, hepatitis, gastritis, constipation	[50]
	Northeastern region	Sidr, Nabq	Barks fruits, leaves, roots	-	-	Hair parasites, gastritis, sciatica, abscess, reinforcement and activation of piles hepatitis, psychiatric and spiritual counseling abdominal issues, constipation	[51,96]
Jordan	The central mountains (North–South) in Jordan	Sader, Orkod	Fruits	Edible	-	Cough and measles	[52]
	Jordan (countryside, and desert)	Ceder	Fruits, seeds	Decoction	-	Vermifuge and antispasmodic	[53]
	-	-	Bark, branches, gum, leaves, root	Powder	-	Toothache	[97]
			Leaves	Powder	-	Cleaning dead bodies. Women utilize leaf powder, which works extremely well as shampoo, in combination with hot water to wash their hair.	
				Decoction	-	Head lice	
				-	-	Treat dandruff and counter obesity	
			-	-	-	Anti-diabetic, antibacterial, anti-cancer, anti-hypertensive, anti-nociceptive, anti-diarrheal, intestinal ailments, colds, and skin.	
Palestine	West Bank	Lotus jujube, zyzafun	Leaves	Decoction	-	Diarrhea	[98]
Cyprus	North Cyprus	Gonnara	Fruits	Raw	-	-	[99]
Nigeria	Kebbi State (northwest)	Tsada	Bark, root	-	-	Cancer	[100]

**Table 2:** Phytoconstituents of the *Z. lotus* extracts.

Country	Used Part	Used Extract	Chemical Compounds	References
Tunisia	Roots	Petroleum ether extract	Ethyl tridecanoate; 2-pentadecanone; pentadecanoic acid, ethyl ester; 13-epimanool; tetradecanoic acid; *n*-hexadecanoic acid	[25]
		Dichloromethane extract	Ethyl tridecanoate; tetradecanoic acid, ethyl ester; 13-epimanool	
	Leaves	Methanol extract	Luteolin; trans cinnamic acid; quercetin; rutin; gallic acid; syringic acid; 4-*O*-caffeoylquinic acid; epicatechin; *trans*-ferulic acid; hyperoside; *p*-coumaric acid; quercitrin; naringin; kaempferol; naringenin; apigenin; acacetin; quinic acid; (+)-catechin	[19]
	Fruits		Quinic acid; luteolin-7-*O*-glucoside; rutin; epicatechin; *p*-coumaric acid; apigenin-7-*O*-glucoside; quercitrin; naringin; chlorogenic acid; 1,3-di-*O*-caffeoylquinic acid; cirsilineol	
	Seeds		Quinic acid; catechin (+); 4,5-di-*O*-caffeoylquinic acid; chlorogenic acid; syringic acid; hyperoside; rutin; 3,4-di-*O*-caffeoylquinic acid; quercitrin; naringin; apigenin-7-*O*-glucoside; 4-*O*-caffeoylquinic acid; trans cinnamic acid; quercetin; *p*-coumaric acid; luteolin; naringenin; apigenin	
	Leaves, and flowers	Essential oil	Nonanal; decanal; linalool; α-cadinol; azulol; farnesyl acetone; 2-undecanone; 2-pentadecanone; tetradecanoic acid, ethyl ester; α-farnesene; carvone; γ-cadinene; tridecanal; trans-*β*-ionone; D-nerolidol; E-nerolidol; hexyl-benzoate; hexahydrofarnesyl acetone; cis-hexenyl-3-benzoate; cadalene; dodecanoic acid; tetradecanoic acid; decanoic acid, ethyl ester; undecanoic acid, ethyl ester; 2-tridecanone; β-cyclocitral; L-α-terpineol; dodecanoic acid, ethyl ester; ethyl tridecanoate; ledol; pentadecanoic acid, ethyl ester; hexadecanoic acid, ethyl ester; damascenone; geranylacetone; α-calacorene	[107]
	Fruits	Methanolic extract	Lauric acid; myristic acid; pentadecyclic acid; palmitic acid; heptaguaric acid; oleic acid; elaidic acid; linoleic acid; α-linolenic; stearidonic acid; arachidic acid; behenic acid; erucic acid	[27]
	Leaves	Methanolic extract	Fumaric acid; catechin; tyrosol; gallic acid; syringic acid; *p*-coumaric acid; vanillin; ferulic acid; caffeic acid; cinnamic acid	[17]
	Leaves	Acetonic extract	Rutin; luteolin-7-*O*-glucoside; naringin; luteolin; kaempferol	[105]
	Leaves	Acetonic extract	Quinic acid; gallic acid; protocatchuic acid; catechin (+); quercetin-3-*O*-galactoside; 4-*O*-caffeoylquinic acid; syringic acid; epicatechin; *p*-coumaric acid; rutin; quercetin-3-*O*-rhamonoside; quercetin; kaempherol; naringenin; apegenin; luteolin	[104]
	Root bark	Classical acid–base method (Alkaloid extract)	Lotusine A; lotusine D	[110]
	Root bark	Classical acid–base method (Alkaloid extract)	Lotusine B; lotusine C; lotusine E; lotusine F	[109]
	Root bark	Classical acid–base method (Alkaloid extract)	Lotusine G	[108]
Morocco	Seeds	Essential oil	Oleic acid; palmitic acid; linoleic acid; stearic acid	[106]
	Fruits	Ethanol extract	Synapic acid; benzoic acid; *p*-coumaric acid; *p*-hydroxybenzoic acid; *p*-coumaroyl glucose; cinnamic acid derivative	[24]
		Methanol extract	Malic acid; (-)-catechin 3-*O*-gallate; quercetin; galloyl shikimic acid; quercetin di-glucoside; rhamnosyl-rhamnosylglucoside; eriodictyol	
	Leaves	Aqueous extract	Gallic acid; catechin; rutin; *p*-hydroxybenzoic acid; caffeic acid; vanillic acid; epicatechin; resveratrol; syringic acid; *p*-coumaric acid; 3-hydroxycinnamic acid; pyrogallol; salicylic acid; naringin; ferulic acid; chlorogenic acid; sinapic acid; rosmarinic acid; quercetin	[20]
	Fruits		Gallic acid; chlorogenic acid; catechin; rutin; *p*-hydroxybenzoic acid; 3-hydroxycinnamic acid; vanillic acid; epicatechin; caffeic acid; syringic acid; *p*-coumaric acid; ferulic acid; pyrogallol; sinapic acid; naringin; salicylic acid; rosmarinic acid; resveratrol; catechol	
	Fruits	Methanolic extract	Macrocarpon C; isovitexin-2″-*O*-rhamnoside; amorfrutin A; hyperin; astragalin	[101]
	Leaves		7,8-Dihydrobiopterin; quercetin-3-galactoside; kaempferol-3-diglucoside	
	Pulp	Dichloromethane extract	Decanoic acid; tridecanoic acid; tetradecanoic acid;; heptadecanoic acid; octadecanoic acid; nonadecanoic acid; eicosanoic acid; heneicosanoic acid; hexacosanoic acid; heptacosanoic acid; octacosanoic acid; triacontanoic acid; tetradecenoic acid; hexadecenoic acid; heptadecenoic acid; (9Z,12Z)-octadeca-9,12-dienoic acid; (9Z,12Z,15Z)-octadeca-9,12,15-trienoic acid; (9Z)-octadec-9-enoic acid; (9E)-octadec-9-enoic acid; nonadecenoic acid; pentadecanoic acid; hexadecanoic acid; undecanoic acid; dodecanoic acid; eicos-11-enoic acid; hexadecanedioic acid; 22-hydroxydocosanoic acid; 2-hydroxytetracosanoic acid; ethyl decanoate; ethyl tetradecanoate; ethyl pentadecanoate; ethyl hexadec-9-enoate; docosanoic acid; pentacosanoic acid; ethyl hexadecanoate; ethyl (9Z)-octadec-9-enoate; ethyl (9E)-octadec-9-enoate; ethyl octadecanoate; ethyl eicosanoate; methyl hexadecanoate; 1-palmitoylglycerol; 1-oleoylglycerol; hexadecan-1-ol; (9Z)-octadec-9-en-1-ol; oleanolic acid; betulinic acid; ursolic acid; stigmasterol; *β*-sitosterol; benzoic acid; vanillic acid; *p*-coumaric acid; solerol; glycerol; octacosanal; nonacosan-10-one; triacontanal	[102]
	Seeds		Decanoic acid; dodecanoic acid; tetradecanoic acid; pentadecanoic acid; hexadecanoic acid; heptadecanoic acid; octadecanoic acid; eicosanoic acid; docosanoic acid; hexadecenoic acid; heptadecenoic acid; (9Z,12Z)-octadeca-9,12-dienoic acid; (9Z)-octadec-9-enoic acid; (9E)-octadec-9-enoic acid; eicos-11-enoic acid; ethyl hexadecanoate; ethyl (9Z)-octadec-9-enoate; methyl (9Z)-octadec-9-enoate; 2-palmitoylglycerol; 1-palmitoylglycerol; 1-linoleoylglycerol; 1-oleoylglycerol; 1-stearoylglycerol; hexadecan-1-ol; oleanolic acid; betulinic acid; ursolic acid; stigmasterol; *β*-sitosterol; benzoic acid; vanillin; vanillyl alcohol; E-ferulic acid; glycerol; squalene	
	Leaves		Decanoic acid; dodecanoic acid; hexadecanoic acid; heptadecanoic acid; octadecanoic acid; eicosanoic acid; heneicosanoic acid; docosanoic acid; tetracosanoic acid; pentacosanoic acid; octacosanoic acid; hexadecenoic acid; (9Z,12Z)-octadeca-9,12-dienoic acid; tetradecanoic acid; pentadecanoic acid; (9Z,12Z,15Z)-octadeca-9,12,15-trienoic acid; (9Z)-octadec-9-enoic acid; (9E)-octadec-9-enoic acid; eicos-11-enoic acid; 22-hydroxydocosanoic acid; 2-palmitoylglycerol; 1-palmitoylglycerol; 1-linoleoylglycerol; 1-linolenoylglycerol; 1-stearoylglycerol; hexadecan-1-ol; (9Z)-octadec-9-en-1-ol; octadecan-1-ol; lupeol; oleanolic acid; betulinic acid; campesterol; *β*-Sitosterol; benzoic acid; salicylic acid; vanillic acid; *p*-coumaric acid; glycerol; loliolide; neophytadiene; inositol; phytol; squalene; *γ*-tocopherol; tetracosyl acetate; α-tocopherol	
	Root Bark		Decanoic acid; dodecanoic acid; heptadecanoic acid; nonadecanoic acid; eicosanoic acid; docosanoic acid; tetracosanoic acid; pentacosanoic acid; hexadecenoic acid; heptadecenoic acid; (9Z,12Z)-octadeca-9,12-dienoic acid; (9Z,12Z,15Z)-octadeca-9,12,15-trienoic acid; (9Z)-octadec-9-enoic acid; octadecanoic acid; (9E)-octadec-9-enoic acid; eicos-11-enoic acid; 22-hydroxydocosanoic acid; tricosanoic acid; heneicosanoic acid; methyl (9Z)-octadec-9-enoate; 1-palmitoylglycerol; 1-linoleoylglycerol; pentadecanoic acid; hexadecanoic acid; 1-oleoylglycerol; 1-stearoylglycerol; tetradecan-1-ol; hexadecan-1-ol; octadecan-1-ol; lupeol; oleanolic acid; betulinic acid; campesterol; stigmasterol; *β*-Sitosterol; vanillin; vanillyl alcohol; syringaldehyde; homovanillyl alcohol; vanillic acid; hydroxytyrosol; protocatechuic acid; syringic acid; glycerol	
	Fruits	Aqueous extract	3-hydroxycinnamic acid; catechin; hydroxytyrosol; naringenin; *p*-coumaric acid; quercetin; rutin; vanillic acid; ferulic acid; gallic acid	[21]
Algeria	Branches	Decoction	Catechin; quercetin-3-*O*-rutinoside; apigenin-*O*-hexoside-*O*-deoxyhexoside; eriodictyol-*O*-deoxyhexoside; oleuropein; quercetin-*O*-deoxyhexoside; oleuropein hexoside	[26]
		Infusion	Catechin; quercetin-3-*O*-rutinoside; apigenin-*O*-hexoside-*O*-deoxyhexoside; eriodictyol-*O*-deoxyhexoside; oleuropein; quercetin-*O*-deoxyhexoside; oleuropein hexoside	
		Hydroethanolic extract	Catechin; quercetin-3-*O*-rutinoside; oleuropein hexoside; apigenin-*O*-hexoside-*O*-deoxyhexoside; eriodictyol-*O*-deoxyhexoside; oleuropein; quercetin-*O*-deoxyhexoside	
	Leaves	Decoction	Quercetin-3-*O*-(2,6-di-*O*-rhamnosylglucoside)-7-*O*-rhamnoside; myricetin-3-*O*-rutinoside; quercetin-3-*O*-(2,6-di-orhamnosylglucoside-7-*O*-glucuronide; kaempferol-3-*O*-(2,6-di-o rhamnosylglucoside); phloretin-di-c-hexoside; kaempferol-*O*-hexoside; kaempferol-3-*O*-(2,6-di-*O*-rhamnosylglucoside); oleuropein hexoside; kaempferol-3-*O*-rutinoside; kaempferol-3-*O*-(6-*O*-rhamnosyl-glucoside); apigenin-*O*-hexoside-*O*-deoxyhexoside; quercetin-3-*O*-rutinoside; oleuropein; quercetin-3-*O*-(2,6-di-*O*-rhamnosylglucoside)	
		Infusion	Quercetin-3-*O*-(2,6-di-*O*-rhamnosylglucoside)-7-*O*-rhamnoside; apigenin-*O*-hexoside-*O*-deoxyhexoside; myricetin-3-*O*-rutinoside; quercetin-3-*O*-(2,6-di-orhamnosylglucoside-7-*O*-glucuronide; kaempferol-3-*O*-(2,6-di-*O*-rhamnosylglucoside); phloretin-di-c-hexoside; quercetin-3-*O*-rutinoside; quercetin-3-*O*-(2,6-di-*O*-rhamnosylglucoside); kaempferol-*O*-hexoside; kaempferol-3-*O*-(2,6-di-*O*-rhamnosylglucoside); oleuropein hexoside; kaempferol-3-*O*-rutinoside; kaempferol-3-*O*-(6-*O*-rhamnosyl-glucoside); oleuropein	
		Hydroethanolic extract	Quercetin-3-*O*-(2,6-di-*O*-rhamnosylglucoside)-7-*O*-rhamnoside; myricetin-3-*O*-rutinoside; quercetin-3-*O*-(2,6-di-orhamnosylglucoside-7-*O*-glucuronide; kaempferol-3-*O*-(2,6-di-*O*-rhamnosylglucoside); phloretin-di-c-hexoside; quercetin-3-*O*-rutinoside; kaempferol-*O*-hexoside; kaempferol-3-*O*-(2,6-di-*O*-rhamnosylglucoside); oleuropein hexoside; kaempferol-3-*O*-rutinoside; kaempferol-3-*O*-(6-*O*-rhamnosyl-glucoside); apigenin-*O*-hexoside-*O*-deoxyhexoside; oleuropein; quercetin-3-*O*-(2,6-di-*O*-rhamnosylglucoside)	
	Root barks	Decoction	(Epi)catechin-(epi)gallocatechin; (+)-catechin; (-)-epicatechin; myricetin-3-*O*-rutinoside	
		Infusion	(Epi)catechin-(epi)gallocatechin; (-)-epicatechin; myricetin-3-*O*-rutinoside	
		Hydroethanolic	(Epi)catechin-(epi)gallocatechin; (+)-Catechin; (-)-Epicatechin; Myricetin-3-*O*-rutinoside	
	Stem barks	Decoction	Oleoside; eriodictyol-*O*-hexoside; quercetin-*O*-deoxyhexoside; eriodictyol-*O*-pentoside; eriodictyol-*O*-deoxyhexoside; eriodictyol-*O*-deoxyhexoside	
		Infusion	Oleoside; quercetin-*O*-deoxyhexoside; eriodictyol-*O*-pentoside; eriodictyol-*O*-deoxyhexoside; eriodictyol-*O*-deoxyhexoside	
		Hydroethanolic	Oleoside; eriodictyol-*O*-hexoside; quercetin-*O*-deoxyhexoside; eriodictyol-*O*-pentoside; eriodictyol-*O*-deoxyhexoside; eriodictyol-*O*-deoxyhexoside	

**Table 3:** The antimicrobial results of the *Z. lotus* extracts.

Used Parts	Extracts	Bacteria or Fungi (Concentration)	References
Leaves	Acetonic extract	*S. aureus* (MIC = 1000 µg/mL; MBC = 2000 µg/mL), *S. aureus* methicillin-resistant (MIC = 250 µg/mL; MBC = 2000 µg/mL), *S. epidermidis* (MIC = 250 µg/mL; MBC = 500 µg/mL), *S. epidermidis* methicillin-resistant (MIC = 500 µg/mL; MBC = 1000 µg/mL), *L. monocytogenes* (MIC = 500 µg/mL; MBC = 2000 µg/mL)	[105]
Pulps	Lipophilic extract	*E. coli* (MIC >2048 µg/mL), *S. aureus* (MIC >2048 µg/mL), *S. epidermidis* (MIC > 2048 µg/mL)	[102]
Seeds		*E. coli* (MIC >2048 µg/mL), *S. aureus* (MIC >2048 µg/mL), *S. epidermidis* (MIC = 1024 µg/mL)	
Leaves		*E. coli* (MIC = 1024 µg/mL), *S. aureus* (MIC = 2048 µg/mL), *S. epidermidis* (MIC = 1024 µg/mL)	
Root bark		*E. coli* (MIC >2048 µg/mL), *S. aureus* (MIC = 2048 µg/mL), *S. epidermidis* (MIC = 2048 µg/mL)	
Leaves	Methanolic extract	*S. aureus* (10 mg/mL; IZ = 12–13 mm), *L. monocytogenes* (10 mg/mL; IZ = 10–12.2 mm), *S. typhimurium* (10 mg/mL; IZ = 11–12.2 mm), *E. coli* (10 mg/mL; IZ = 10.6–11.8 mm)	[19]
Seeds	Ethanolic extract	*E. coli* (MIC = 50 mg/mL), *P. aeruginosa* (MIC = 50 mg/mL), *S. aureus* (MIC = 100 mg/mL), *E. faecalis* (MIC = 50 mg/mL)	[18]
	Methanolic extract	*E. coli* (MIC = 100 mg/mL), *P. aeruginosa* (MIC = 50 mg/mL), *S. aureus* (MIC = 100 mg/mL), *E. faecalis* (MIC = 50 mg/mL)	
	Aqueous extract	*E. coli* (MIC = 200 mg/mL), *P. aeruginosa* (MIC = 100 mg/mL), *S. aureus* (MIC = 200 mg/mL), *E. faecalis* (MIC = 100 mg/mL)	
Stems	Methanolic extract	*S. aureus* (MIC = 7 mg/mL), *E. coli* (MIC = 6 mg/mL), *P. aeruginosa* (MIC = 6 mg/mL)	[133]
Fruits	Methanol extract	*E. coli* (MIC = 400 μg/mL), *Agrobacterium* sp (MIC = 400 μg/mL), *Rhizobium* sp (MIC = 3.2 μg/mL), *B. pumilus* (MIC = 320 μg/mL), *B. subtilis* (MIC = 340 μg/mL)	[134]
Leaves	Methanolic extract	*B. subtilis* (MIC = 12.5 μg/mL), *S. aureus* (MIC = 25.0 μg/mL), *E. coli* (MIC = 1000 μg/mL), *P. aeruginosa* (MIC = 1000 μg/mL), *S. Typhimurium* (MIC = 1000 μg/mL)	[135]
Fruits	Ethanolic extract	*E. coli* (MIC = 50 mg/mL), *P. aeruginosa* (MIC = 25 mg/mL), *S. aureus* (MIC = 25 mg/mL), *S. epidermidis* (MIC = 25 mg/mL), *E. faecalis* (MIC = 25 mg/mL), *B. subtilis* (MIC = 25 mg/mL), *M. luteus* (MIC = 25 mg/mL)	[136]
	Methanolic extract	*E. coli* (MIC = 50 mg/mL), *P. aeruginosa* (MIC = 25 mg/mL), *S. aureus* (MIC = 25 mg/mL), *S. epidermidis* (MIC = 25 mg/mL), *E. faecalis* (MIC = 25 mg/mL), *B. subtilis* (MIC = 25 mg/mL), *M. luteus* (MIC = 25 mg/mL), *C. tropicalis* (MIC = 50 mg/mL)	
	Aqueous extract	*E. coli* (MIC = 100 mg/mL), *P. aeruginosa* (MIC = 200 mg/mL), *S. aureus* (MIC = 12.5 mg/mL), *S. epidermidis* (MIC = 200 mg/mL), *E. faecalis* (MIC = 200 mg/mL), *B. subtilis* (MIC = 100 mg/mL), *M. luteus* (MIC = 100 mg/mL), *C. tropicalis* (MIC = 100 mg/mL)	
Fruits	Methanolic extract	*H. pylori* (MIC = 128 μg/mL)	[117]

**Table 4:** The anti-oxidant activities of the *Z. lotus* extracts.

Country	Region	Used Part	Extract	Method	Results	References
Morocco	Northeastern	Fruits	AqE	DPPH	IC_50_ = 116 ± 0.02 µg/mL	[35]
				*β*-carotene bleaching test	12.5 µg/mL (42.24% of oxidation), 25 µg/mL (31.68% of oxidation), 50 µg/mL (26.92% of oxidation), and 100 µg/mL (21.11% of oxidation)	
	Region of Ihahen (southern region)	Fruits	HxE	DPPH	IC_50_ = 8 mg/mL	[101]
			MtOH		IC_50_ = 5 mg/mL	
			DiMtn		IC_50_ > 10 mg/mL	
		Leaves	HxE		IC_50_ > 40 mg/mL	
			MtOH		IC_50_ = 0.7 mg/mL	
			DiMtn		IC_50_ > 40 mg/mL	
	Fez (Zouagha-Moulay Yaâcoub)	Seeds	MtOH	DPPH	IC_50_ = 1.33 ± 0.01 mg/mL	[18]
			EtOH		IC_50_ = 1.32 ± 0.09 mg/mL	
			AqE		IC_50_ = 3.11 ± 0.05 mg/mL	
	Region of Sidi Sliman	Fruits	AqE	DPPH	74.87 ± 16.74 mg TE/g EDW	[20]
				ABTS	46.31 ± 11.02 mg TE/g EDW	
				FRAP	55.30 ± 2.30 mg AAE/g EDW	
		Leaves		DPPH	241.75 ± 17.37 mg TE/g EDW	
				ABTS	301.34 ± 8.26 mg TE/g EDW	
				FRAP	160.10 ± 2.30 mg AAE/g EDW	
	Zaouiat Cheikh Area, Oued Zem City	Fruits	MtOH	DPPH	IC_50_ = 131.01 µg/mL	[134]
				ABTS	IC_50_ = 52.42 µg/mL	
Tunisia	Oudhref-Gabes Region (South of Tunisia)	Roots	PeE	ABTS	IC_50_ = 14.76 ± 0.02 mg/L	[25]
			DiMtn	ABTS	IC_50_ = 136.58 ± 0.41 mg/L	
			MtOH		IC_50_ = 14.31 ± 0.13 mg/L	
			EtOH		IC_50_ = 27.42 ± 0.32 mg/L	
			AqE		IC_50_ = 8.96 ± 0.38 mg/L	
			PeE	DPPH	IC_50_ = 101.06 ± 0.40 mg/L	
			DiMtn		IC_50_ = 192.33 ± 0.60 mg/L	
			MtOH		IC_50_ = 18.03 ± 0.61 mg/L	
			EtOH		IC_50_ = 39.50 ± 0.49 mg/L	
			AqE		IC_50_ = 16.46 ± 0.60 mg/L	
			PeE	TAC	105.56 ± 0.37 mg AAE/mg EDW	
			DiMtn		91.11 ± 2.20 mg AAE/mg EDW	
			MtOH		304.07 ± 1.11 mg AAE/mg EDW	
			EtOH		167.41 ± 7.40 mg AAE/mg EDW	
			AqE		191.85 ± 0.00 mg AAE/mg EDW	
		Leaves	PeE	ABTS	IC_50_ = 28.98 ± 0.06 mg/L	
			DiMtn		IC_50_ = 29.51 ± 1.23 mg/L	
			MtOH		IC_50_ = 23.48 ± 0.63 mg/L	
			EtOH		IC_50_ = 249.37 ± 1.26 mg/L	
			AqE		IC_50_ = 29.01 ± 0.44 mg/L	
			PeE	DPPH	Not active	
			DiMtn		Not active	
			MtOH		IC_50_ = 33.66 ± 0.11 mg/L	
			EtOH		IC_50_ = 375.50 ± 1.50 mg/L	
			AqE		IC_50_ = 64.80 ± 0.36 mg/L	
			PeE	TAC	Not active	
			DiMtn		154.44 ± 6.20 mg AAE/mg EDW	
			MtOH		142.47 ± 0.85 mg AAE/mg EDW	
			EtOH		173.09 ± 2.99 mg AAE/mg EDW	
			AqE		99.26 ± 4.62 mg AAE/mg EDW	
		Fruits	MtOH	ABTS	IC_50_ = 173.93 ± 0.88 mg/L	
			AqE		IC_50_ = 342.25 ± 1.25 mg/L	
			MtOH	DPPH	IC_50_ = 343.00 ± 1.32 mg/L	
			AqE		IC_50_ = 383.33 ± 0.29 mg/L	
			MtOH	TAC	26.42 ± 2.26 mg AAE/mg EDW	
			AqE		40.74 ± 3.39 mg AAE/mg EDW	
	Tozeur (South of Tunisia)	Fruits	EtOH	TAC	75.981 mg GAE/g EDW	[141]
				DPPH	0.289 mg/mL	
	Region Sidi Aich in the south	Leaves	MtOH	DPPH	IC_50_ = 1.28 ± 0.13 mg/mL	[142]
				FRAP	IC_50_ = 2.18 ± 0.05 mg/mL	
	Kairouan	Leaves	AqE	DPPH	IC_50_ = 0.4 µg/mL	[143]
			EtOH		IC_50_ = 0.1 µg/mL	
	Rouhia		AqE		IC_50_ = 0.6 µg/mL	
			EtOH		IC50 = 0.03 µg/mL	
	Mahres		AqE		IC_50_ = 0.55 µg/mL	
			EtOH		IC_50_ = 0.46 µg/mL	
	Mahdia		AqE		IC_50_ = 0.64 µg/mL	
			EtOH		IC_50_ = 0.35 µg/mL	
	Bengardane	Leaves	MtOH	TAC	30.95 ± 0.01 mg GAE/g EDW	[19]
				DPPH	IC_50_ = 18.27 ± 0.28 µg/mL	
		Fruits		TAC	23.87 ± 0.34 mg GAE/g EDW	
				DPPH	IC_50_ = 12.16 ± 0.31µg/mL	
		Seeds		TAC	22.03 ± 3.08 mg GAE/g EDW	
				DPPH	IC_50_ = 18.57 ± 6.67µg/mL	
	Oued Esseder	Leaves	MtOH	TAC	30.91 ± 0.06 mg GAE/g EDW	
				DPPH	IC_50_ = 16.60 ± 1.58 µg/mL	
		Fruits		TAC	25.02 ± 0.55 mg GAE/g EDW	
				DPPH	IC_50_ = 15.15 ± 0.90 µg/mL	
		Seeds		TAC	22.80 ± 0.15 mg GAE/g EDW	
				DPPH	IC_50_ = 11.41 ± 0.35 µg/mL	
Algeria	Not defined	Fruits	AqE	DPPH	IC_50_ = 11–30 µg/mL	[114]
	Steppic Region of Tiaret	Stems	MtOH	DPPH	480.20 ± 40.64 mg AAE/g EDW	[133]
Italy	Addaura (the northern slopes of Monte Pellegrino, Palermo, Italy)	Stem bark	MtOH	DPPH	304.02 ± 4.80 mg AAE/g EDW	[140]
				Metal chelating	39.01 ± 4.30 mg EDTAE/g EDW	
				FRAP	296.68 ± 1.81 mg TE/g EDW	

**Abbreviations:** DPPH: 2-2-diphenyl-1-picrylhydrazyl; FRAP: ferric reducing/antioxidant power; TAC: total antioxidant capacity; ABTS: 2,2′-azino-bis(3-ethylbenzothiazoline-6-sulfonic acid); TE: Trolox equivalent; EDW: extract dry weight; AAE: ascorbic acid equivalent; GAE: gallic acid equivalent; EDTAE: ethylenediaminetetraacetic acid equivalent; IC_50_: median inhibitory concentration. Extracts: AqE: aqueous extract; HxE: hexane extract; MtOH: methanol extract; DiMtn: dichloromethane extract; EtOH: ethanol extract; PeE: petroleum ether extract.

## Data Availability

Not applicable.

## Abbreviation

LC-ESI–MS: liquid chromatography-electrospray ionization-tandem mass spectrometry, GC-MS: gas chromatography-mass spectrometry, HPLC: high-performance liquid chromatography, MTT assay: 3-(4,5-dimethylthiazol-2-yl)-2,5-diphenyltetrazolium bromide assay, GM: gentamicin, CCl_4_: carbon tetrachloride, AST: aspartate aminotransferase, ALT: alanine aminotransferase, ALP: alkaline phosphatase, *Z. lotus*: *Ziziphus lotus* (L.) Lam., CaOx: calcium oxalate, CaCo-2: carcinoma, K-562: myeloid leukemia, MIC: minimum inhibitory concentration, MBC: minimum bactericidal concentration, DPPH: 2-2-diphenyl-1-picrylhydrazyl; FRAP: ferric reducing/antioxidant power; TAC: total antioxidant capacity; ABTS: 2,2′-azino-bis(3-ethylbenzothiazoline-6-sulfonic acid); TE: Trolox equivalent; EDW: extract dry weight; AAE: ascorbic acid equivalent; GAE: gallic acid equivalent; EDTAE: ethylenediaminetetraacetic acid equivalent; IC_50_: median inhibitory concentration, ERK1/2: 1/2 kinase controlled by the extracellular signal, IL-2: interleukin-2, MTT assay: 3-(4,5-dimethylthiazol-2-yl)-2,5-diphenyltetrazolium bromide assay, DNA: deoxyribonucleic acid, CYP: cypermethrin.
